# Assessment of simplified ratio-based approaches for quantification of PET [^11^C]PBR28 data

**DOI:** 10.1186/s13550-017-0304-1

**Published:** 2017-07-21

**Authors:** Granville J. Matheson, Pontus Plavén-Sigray, Anton Forsberg, Andrea Varrone, Lars Farde, Simon Cervenka

**Affiliations:** 1 Department of Clinical Neuroscience, Centre for Psychiatry Research, Karolinska Institutet and Stockholm Health Care Services, Stockholm County Council, Stockholm, Sweden; 2 Personalised Healthcare and Biomarkers, AstraZeneca PET Science Centre, Karolinska Institutet, Stockholm, Sweden; 3 Department of Clinical Neuroscience, Karolinska Institutet, Karolinska University Hospital, R5:00, SE-171 76 Stockholm, Sweden

**Keywords:** PET, TSPO, [^11^C]PBR28, SUVR, DVR

## Abstract

**Purpose:**

Kinetic modelling with metabolite-corrected arterial plasma is considered the gold standard for quantification of [^11^C]PBR28 binding to the translocator protein (TSPO), since there is no brain region devoid of TSPO that can serve as reference. The high variability in binding observed using this method has motivated the use of simplified ratio-based approaches such as standardised uptake value ratios (SUVRs) and distribution volume (V_T_) ratios (DVRs); however, the reliability of these measures and their relationship to V_T_ have not been sufficiently evaluated.

**Methods:**

Data from a previously published [^11^C]PBR28 test-retest study in 12 healthy subjects were reanalysed. V_T_ was estimated using a two-tissue compartment model. SUVR and DVR values for the frontal cortex were calculated using the whole brain and cerebellum as denominators. Test-retest reliability was assessed for all measures. Interregional correlations were performed for SUV and V_T_, and principal component analysis (PCA) was applied. Lastly, correlations between ratio-based outcomes and V_T_ were assessed.

**Results:**

Reliability was high for V_T_, moderate to high for SUV and SUVR, and poor for DVR. Very high interregional correlations were observed for both V_T_ and SUV (all *R* ^2^ > 85%). The PCA showed that almost all variance (>98%) was explained by a single component. Ratio-based methods correlated poorly with V_T_ (all *R* ^2^ < 34%, divided by genotype).

**Conclusions:**

The reliability was good for SUVR, but poor for DVR. Both outcomes showed little to no association with V_T_, questioning their validity. The high interregional correlations for V_T_ and SUV suggest that after dividing by a denominator region, most of the biologically relevant signal is lost. These observations imply that results from TSPO PET studies using SUVR or DVR estimates should be interpreted with caution.

## Introduction

The PET radioligand [^11^C]PBR28 binds to the translocator protein (TSPO), which is expressed in glial cells and regarded as a marker of brain immune function. Since there is no reference brain region devoid of TSPO [1], kinetic modelling with metabolite-corrected arterial plasma as input function is considered the gold standard for analysis of [^11^C]PBR28 binding, and the distribution volume (V_T_) is the commonly used outcome measure. There is, however, a large degree of intra- and interindividual variability in V_T_, even after accounting for TSPO affinity genotype [2, 3]. This variability reduces sensitivity for detection of effects in clinical studies. In attempts to circumvent this shortcoming, simplified ratio-based approaches, including standardised uptake value ratios (SUVRs), or distribution volume ratios (DVRs), have been suggested and applied [4–6].

Recently, a test-retest analysis of [^11^C]PBR28 SUVR values in Alzheimer’s disease patients was reported, showing an apparent high utility of this method [7]. The study observed low absolute percentage variability and high intraclass correlation coefficient (ICC) values in five high-affinity binders (HABs). Apart from reducing variability, this approach would additionally be advantageous from a practical perspective, by omitting the need for arterial blood sampling. However, the study did not examine the association of SUVR to traditional V_T_ values. With regard to DVR, neither reliability nor relation to V_T_ has been examined.

The objectives of this study were to assess the test-retest reliability of [^11^C]PBR28 ratio-based outcomes and to examine their association with V_T_ in healthy control subjects. We also investigated the interregional correlations for SUV and V_T_ respectively, since the relationships between target and denominator regions, which both contain TSPO, may influence both the reliability and validity of ratio-based outcome measures.

## Materials and methods

### Subjects

PET measurements from 12 healthy subjects (mean age 23.9, sd 2.99, 6 females) who had participated in a previous test-retest study of [^11^C]PBR28 binding [2] were included in the analysis. Six participants were mixed-affinity binders (MABs) and six were high-affinity binders (HABs). The study was approved by the Karolinska University Hospital Radiation Safety Committee and the Regional Ethics Committee in Stockholm. All subjects gave written informed consent prior to participating.

### Test-retest study design

Six of the subjects underwent the two PET examinations on the same day, and for the remaining six, the examinations were run 2–5 days apart. Radiosynthesis and production of [^11^C]PBR28 was performed as described previously [2]. All examinations were performed using the high-resolution research tomograph (Siemens Molecular Imaging, Knoxville, TN). Radioactivity concentration in blood was obtained by arterial measurements, from which a metabolite-corrected arterial input function was derived as described previously [8].

For one individual, the second PET examination was shortened due to technical issues. This subject was excluded from the test-retest analysis, but the first PET examination was included in correlational analyses.

### Quantification of [^11^C]PBR28 and ratio-based outcomes

Segmentation and ROI delineation of the subjects’ T1-weighted MRI images was performed using FreeSurfer (5.0.0, http://surfer.nmr.mgh.harvard.edu/). Time-activity curves (TACs) were extracted for the whole brain, cerebellar cortex, frontal cortex, temporal cortex, striatum and thalamus.

SUVs were calculated between 40 and 60 min in order to allow for a direct comparison with Nair et al. [7]. To derive V_T_ values, kinetic modelling was performed on TACs from 0 to 63 min, using the R package kinfitr (version 0.2.0,www.github.com/mathesong/kinfitr).

The fractional volume of blood present in the tissue volume (vB) and the delay between the arterial input function and TACs were fitted using the two tissue compartment model (2TCM) and the whole brain TAC. Subsequently, total distribution volume (V_T_) for each ROI was estimated using 2TCM using the fitted delay and vB from the prior whole brain fit. vB values ranged between 2.7 and 6.4% (HABs: mean = 3.9%, sd = 1.0%; MABs: mean = 3.8%, sd = 0.8%).

We used two different denominator regions, i.e. the whole brain (WB) and cerebellum (CBL), to derive the ratio-based outcomes for SUV and V_T_ for FC. This produced the following outcome measures: SUVR_WB_, SUVR_CBL_, DVR_WB_ and DVR_CBL_.

### Statistical analysis

Interregional correlations were derived for ROI V_T_ and SUV values. Principal component analysis (PCA) was used to further examine the correlational structure of the data by identifying the number of independent components required to explain the majority of the variability in ROI V_T_ values. PCAs were performed independently for PET1 and PET2 after *z*-score standardisation of V_T_ values within genotype groups. For the analyses of variability and reliability, we focused on the frontal cortex as target region. The coefficient of variation (COV) was calculated for all outcome measures, using both PET measurements combined. We used the intraclass correlation coefficient (ICC) as a measure of test-retest reliability. The one-way ANOVA fixed effects ICC was used:

$$ ICC=\frac{MS_{\mathrm{B}}-{MS}_{\mathrm{W}}}{MS_{\mathrm{B}}+\left(k-1\right){MS}_{\mathrm{W}}} $$

where MS_B_ and MS_W_ represent the between- and within-subject mean sums of squares and *k* represents the number of groups (in this case 2). We also calculated the absolute percentage variability (VAR), and the standard error of measurement (SEM) (expressed as a ratio to the mean value, providing the estimated within-subject COV) [9]. For V_T_, genotype groups were analysed separately. For ratio methods, results are also reported as combined, since the genotype effect is mostly cancelled out. Finally, we correlated SUV and all ratio-based outcomes against V_T_. All statistical analysis was performed in R (version 3.4.0).

## Results

### Interregional correlations and principal component analysis

**Fig. 1:**
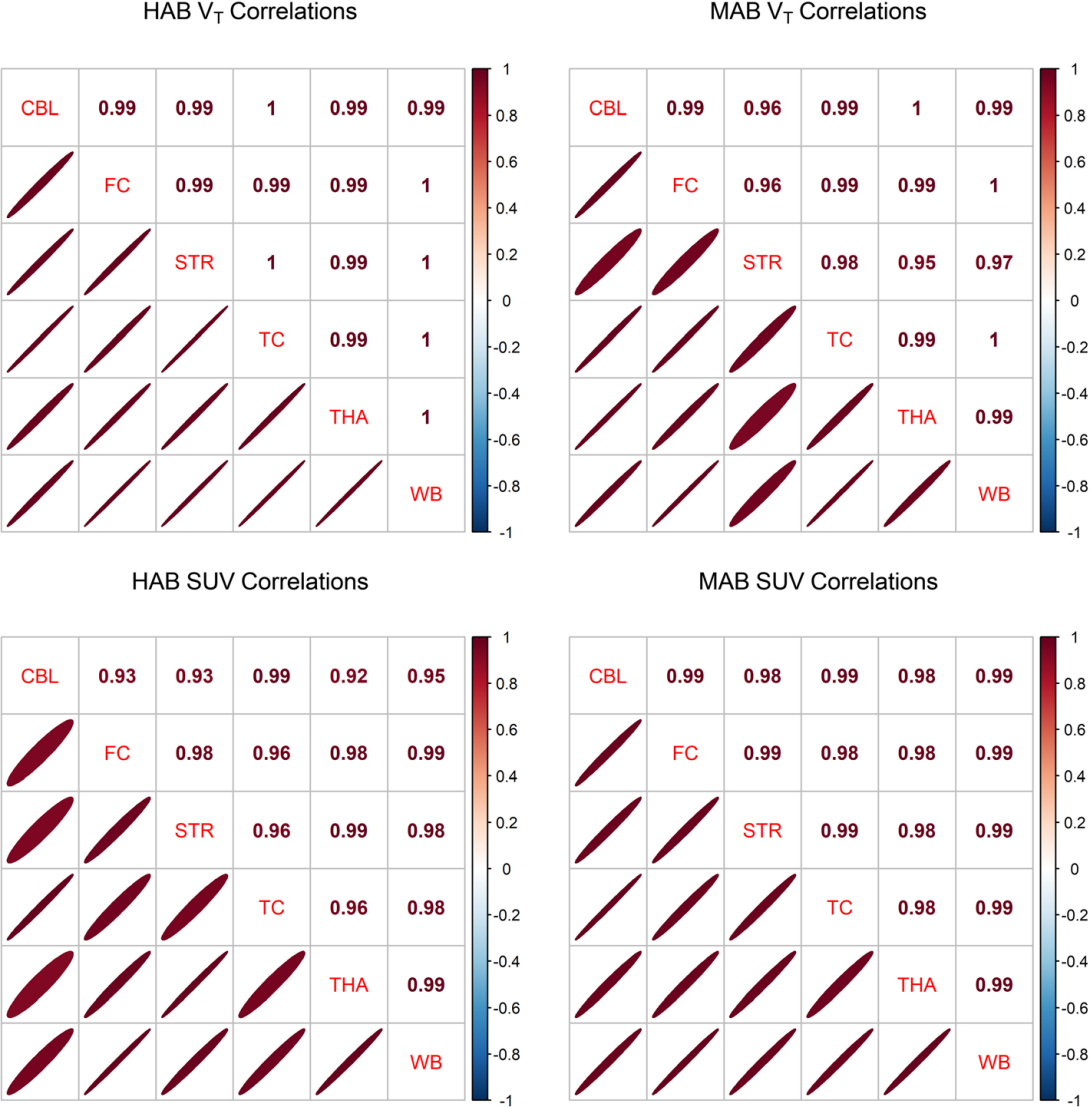
Interregional correlations of [^11^C]PBR28 V_T_ and SUV. Values represent Pearson’s correlation coefficients. *Ellipses* designate the magnitude and the direction of the correlation

The interregional correlations were high both for V_T_ and for SUV, in both HABs and MABs (all *R* ^2^ > 0.85) (Fig. 1).

Using all six ROIs, the first component of the PCAs explained 98.7 and 99.4% of the total variability for PET1 and PET2 respectively. Using only the frontal cortex, whole brain and cerebellum, the first component explained 99.6 and 99.7% respectively.

### Variability and test-retest reliability in the frontal cortex

High inter- and intraindividual variability was observed both for V_T_ and SUV. V_T_ showed high reliability with ICC values of 0.89 (HABs) and 0.93 (MABs), corresponding to 11 and 7% of the variance estimated as being attributable to error respectively. SUV and SUVR showed moderate to high reliability, while DVR showed poor reliability with half on average of the signal estimated to be attributable to error (Table 1).

**Table 1:** Mean values, variability and test-retest metrics for V_T_, SUV, SUVR and DVR using the frontal cortex as the target region and the cerebellum (CBL) or whole brain (WB) as the denominator region for the ratio-based outcomes

Measure	Genotype	Denominator	Mean	COV	ICC	VAR	SEM
V_T-2TCM_	HAB	–	3.9	42	0.89	21	14
V_T-2TCM_	MAB	–	2.2	46	0.93	17	12
V_T-2TCM_	All	CBL	0.98	7.1	0.54	4.7	4.9
V_T-2TCM_	HAB	CBL	0.97	8.1	0.87	3.4	2.9
V_T-2TCM_	MAB	CBL	0.99	6.6	0.17	5.9	6
V_T-2TCM_	All	WB	1	3.6	0.52	3	2.5
V_T-2TCM_	HAB	WB	1.1	3	0.33	3.1	2.4
V_T-2TCM_	MAB	WB	1	3.7	0.55	2.9	2.5
SUV_40_–_60 min_	HAB	–	1.1	22	0.76	13	11
SUV_40_–_60 min_	MAB	–	0.8	31	0.91	13	9.1
SUV_40_–_60 min_	All	CBL	0.95	6.2	0.63	4.4	3.8
SUV_40_–_60 min_	HAB	CBL	0.94	7.5	0.85	3.2	2.9
SUV_40_–_60 min_	MAB	CBL	0.95	5.2	0.32	5.4	4.3
SUV_40_–_60 min_	All	WB	1	3.9	0.89	1.5	1.3
SUV_40_–_60 min_	HAB	WB	1	1.9	0.6	1.3	1.2
SUV_40_–_60 min_	MAB	WB	1	4	0.89	1.6	1.3

*HAB* high-affinity binders, *MAB* mixed-affinity binders, *CBL* cerebellum, *WB* whole brain, *COV* coefficient of variance (%), *ICC* intraclass correlation coefficient, *VAR* absolute percentage variability (%), *SEM* standard error of measurement (%)

### Relationships with V_T_

**Fig. 2:**
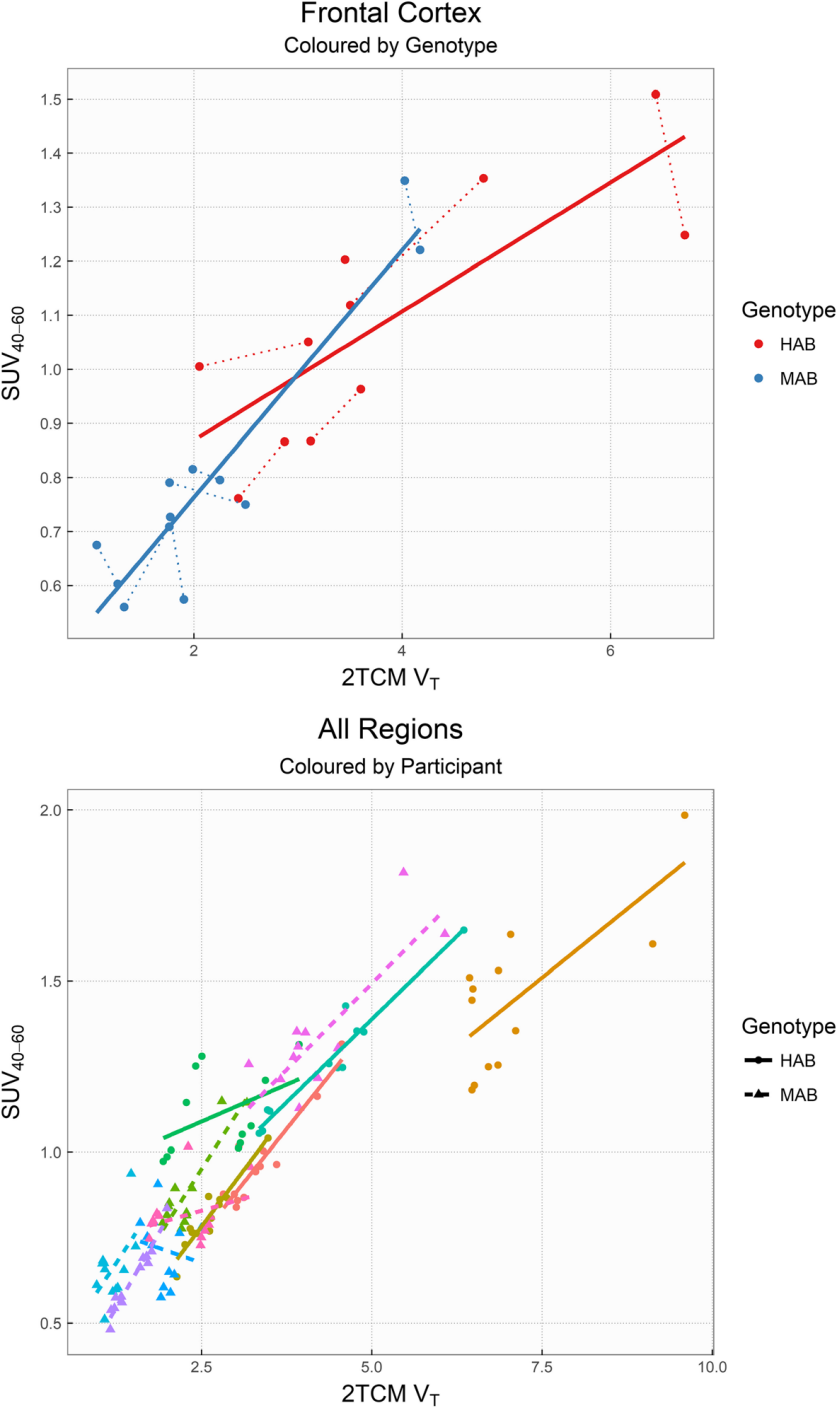
[^11^C]PBR28 V_T_ correlated with SUV, within genotype groups (*upper plot*, frontal cortex) and within subjects (*lower plot*, all brain regions). *Dotted lines* in the *upper plot* indicate repeated measurements. *Solid* and *dashed lines* in the *bottom plot* represent HABs and MABs respectively

**Fig. 3:**
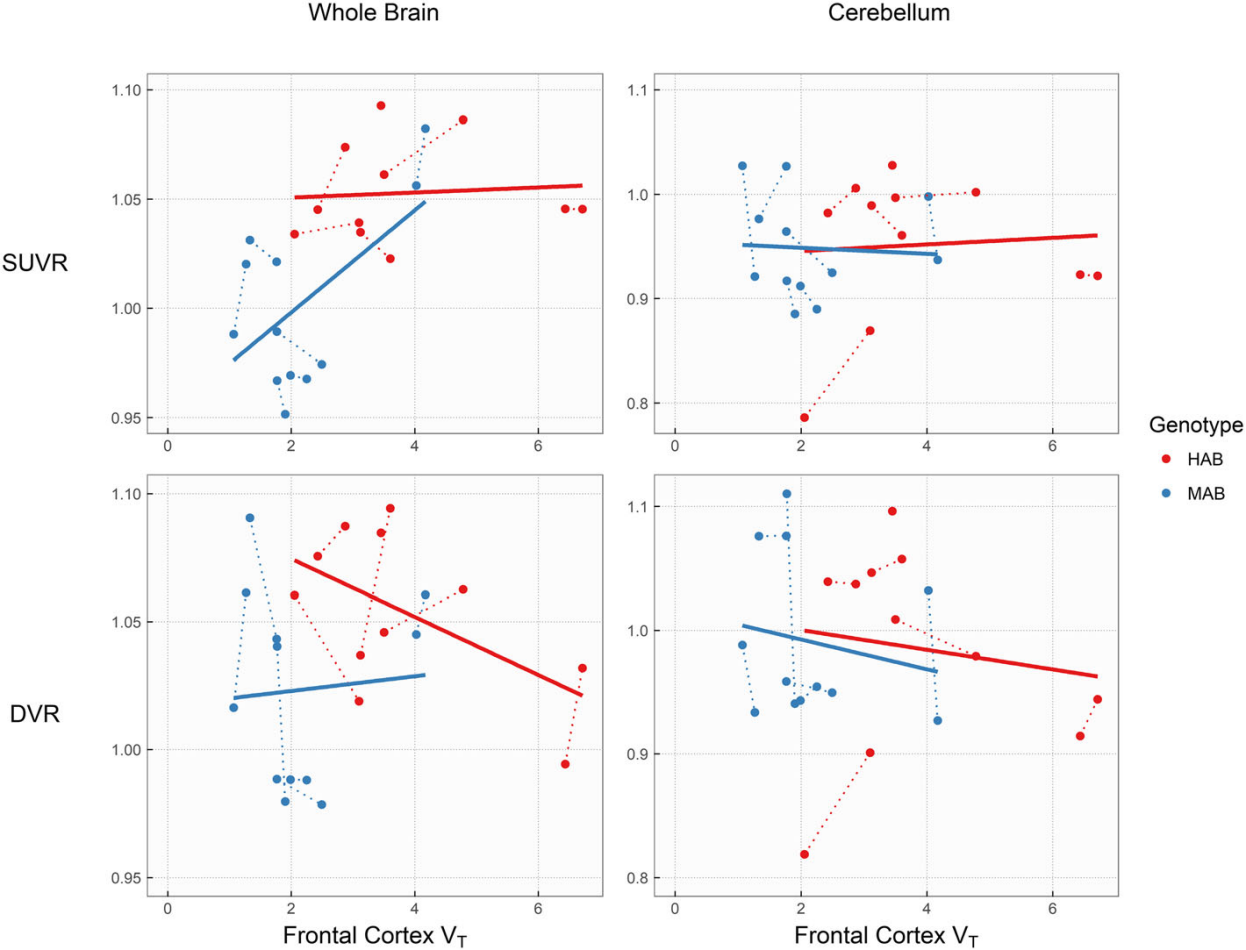
Associations between frontal cortex V_T_ and ratio-based outcomes, using the whole brain and cerebellum as denominator regions. *Dotted lines* indicate repeated measurements

SUV was found to be moderately associated with V_T_, but the estimated association differed both between genotypes, as well as between individuals (Fig. 2). SUVR and DVR correlations with V_T_, after separating individuals by genotype, showed *R* ^2^ values <34% for all regions (Table 2 and Fig. 3).

**Table 2:** Associations with frontal cortex V_T_

Measure	Genotype	*R* ^2^
SUV	HAB	0.64
SUV	MAB	0.86
DVR_CBL_	HAB	0.02
DVR_CBL_	MAB	0.03
DVR_WB_	HAB	0.30
DVR_WB_	MAB	0.00
SUV_CBL_	HAB	0.00
SUV_CBL_	MAB	0.00
SUV_WB_	HAB	0.01
SUV_WB_	MAB	0.33

*HAB* high-affinity binders, *MAB* mixed-affinity binders, *DVR* distribution volume ratio, *SUVR* standard uptake value ratio, *CBL* cerebellum, *WB* whole brain

## Discussion

The reliability of SUVR was moderate to high as has been reported earlier in Alzheimer’s disease patients (7). For DVR, the reliability was poor. For both SUVR and DVR, associations with the traditional outcome measure V_T_ were weak or non-existent. Hence, if V_T_ is considered to be at least moderately associated with brain TSPO levels in healthy subjects, the validity of ratio-based methods must be questioned.

The interregional correlations and PCA showed that almost all variability between ROIs, including denominator ROIs, is attributable to a single underlying dimension of variance. Consequently, dividing the outcome from a target region with a highly correlated denominator region leaves minimal residual differences between individuals. This means that a large part of the biologically relevant signal is lost. This will be the case especially when using the whole brain as a denominator, as the target region is included within the reference region. Although the resulting low COV, VAR and SEM values for SUVR and DVR may seem reassuring, the low reliability as well as the weak correlations with VT does indeed suggest that the remaining variance is largely attributable to noise.

Importantly, this study was conducted using young, healthy participants. As such, no regionally specific alterations in TSPO binding are to be expected, which may partially account for the high degree of interregional correlations observed. The present results suggest that SUVR or DVR estimates may be useful when there is already strong evidence for regionally specific changes in TSPO expression (for example, [10]). These approaches have been suggested also for diseases which affect the brain more globally, based on evidence for a region with relatively spared pathology [4, 7]. However, in practice, the use of ratio methods is conditional on prior knowledge of both (i) significant changes in TSPO expression in target regions such that interregional correlations are reduced and (ii) significant *equivalence* [11, 12] of TSPO expression in the reference region between groups. These prerequisites have, to our knowledge, not yet been fulfilled for any disease or TSPO radioligand, and results obtained using SUVR or DVR estimates should therefore be interpreted with caution. When the whole brain is used as denominator, (i) and (ii) are particularly unlikely to co-occur due to the overlap between target and reference regions. As shown in our analysis, this leads to further reductions in variability which may result in artificially inflated effect sizes, sometimes even in the direction opposite to that of the raw V_T_ values [5].

We found medium to high reliability of SUV, suggesting a potential utility of this method. However, the relationship with V_T_ differed between both genotypes and individuals, which is in line with previous observations in non-human primates [13]. This may indicate a non-linear relationship between SUV and V_T_, in which case a potential ceiling effect may lead to loss of sensitivity of SUV to detect increases in binding. More importantly, the use of SUV relies on the assumption of no differences in radioligand delivery to the brain between groups. In patient-control samples, this is not something that can be safely assumed, since the disorder may involve changes in brain blood flow, and where differences in metabolism, protein binding or peripheral TSPO binding cannot be excluded without arterial sampling.
